# Integrated interventions and supporting activities to increase uptake of multiple cancer screenings: conceptual framework, determinants of implementation success, measurement challenges, and research priorities

**DOI:** 10.1186/s43058-022-00353-8

**Published:** 2022-10-05

**Authors:** Sujha Subramanian, Florence K. L. Tangka, Sonja Hoover, Amy DeGroff

**Affiliations:** 1 RTI International, 307 Waverley Oaks Road, Suite 101, Waltham, MA 02452-8413 USA; 2 Division of Cancer Prevention and Control, National Center for Chronic Disease Prevention and Health Promotion, Centers for Disease Control and Prevention, Atlanta, GA USA

**Keywords:** Integrated interventions, Evidence-based interventions, Multicomponent interventions, Cancer screening

## Abstract

**Background:**

Screening for colorectal, breast, and cervical cancer has been shown to reduce mortality; however, not all men and women are screened in the USA. Further, there are disparities in screening uptake by people from racial and ethnic minority groups, people with low income, people who lack health insurance, and those who lack access to care. The Centers for Disease Control and Prevention funds two programs—the Colorectal Cancer Control Program and the National Breast and Cervical Cancer Early Detection Program—to help increase cancer screenings among groups that have been economically and socially marginalized. The goal of this manuscript is to describe how programs and their partners integrate evidence-based interventions (e.g., patient reminders) and supporting activities (e.g., practice facilitation to optimize electronic medical records) across colorectal, breast, and cervical cancer screenings, and we suggest research areas based on implementation science.

**Methods:**

We conducted an exploratory assessment using qualitative and quantitative data to describe implementation of integrated interventions and supporting activities for cancer screening. We conducted 10 site visits and follow-up telephone interviews with health systems and their partners to inform the integration processes. We developed a conceptual model to describe the integration processes and reviewed screening recommendations of the United States Preventive Services Task Force to illustrate challenges in integration. To identify factors important in program implementation, we asked program implementers to rank domains and constructs of the Consolidated Framework for Implementation Research.

**Results:**

Health systems integrated interventions for all screenings across single and multiple levels. Although potentially efficient, there were challenges due to differing eligibility of screenings by age, gender, frequency, and location of services. Program implementers ranked complexity, cost, implementation climate, and engagement of appropriate staff in implementation among the most important factors to success.

**Conclusion:**

Integrating interventions and supporting activities to increase uptake of cancer screenings could be an effective and efficient approach, but we currently do not have the evidence to recommend widescale adoption. Detailed multilevel measures related to process, screening, and implementation outcomes, and cost are required to evaluate integrated programs. Systematic studies can help to ascertain the benefits of integrating interventions and supporting activities for multiple cancer screenings, and we suggest research areas that might address current gaps in the literature.

**Supplementary Information:**

The online version contains supplementary material available at 10.1186/s43058-022-00353-8.

Contributions to the literature

- The authors provide an overview of implementation science and mixed methods that can be used to evaluate integrated delivery of interventions and supporting activities to promote cancer screening.
- The authors designed a framework to evaluate integrated cancer screening programs, which consists of three categories: multilevel implementation of interventions and supporting activities; screening delivery phases; and evaluation components.
- The authors propose eight areas of research to address current knowledge gaps to ascertain the benefits of integration of interventions and determine best practices.

## Background

Screening for colorectal, breast, and cervical cancer can substantially reduce morbidity and mortality from these cancers [1–3]. In the USA in 2018, 72.4 and 82.9% of women eligible for breast and cervical cancer screening, respectively, were up-to-date with screening recommendations, while 66.9% of eligible adults had undergone colorectal cancer screening [4]. However, these national percentages of screening uptake mask disparities that exist for people from racial and ethnic minority groups, people with low income, people who lack health insurance, and who lack access to care [4–7].

The Centers for Diseases Control and Prevention (CDC) funds two programs that attempt to close the disparities gap in cancer screening. Beginning in 2005, the CDC initiated a longitudinal series of programs to identify optimal approaches to improving the national uptake of colorectal cancer screening [8–10]. The CDC launched the Colorectal Cancer Control Program (CRCCP) in 2009 and the program currently supports states, universities, tribal organizations, and health systems to promote colorectal cancer screening using evidence-based interventions described in The Guide to Community Preventive Services (“Community Guide”) [11]. Furthermore, for 30 years, CDC’s National Breast and Cervical Cancer Early Detection Program (NBCCEDP) has provided access to timely breast and cervical cancer screening and diagnostic services for women who have low incomes and are uninsured and underserved. Many of the health systems are funded by both the CRCCP and NBCCEDP, which offers synergies in planning and evidence-based interventions (e.g., patient reminders and provider reminders) and other supporting activities (e.g., practice facilitation for electronic medical record optimization).

In 2016, CDC created the CRCCP Learning Collaborative to initiate a platform for sharing best practices, a resource to build implementation science research capacity, and a network to support systematic mixed methods evaluations [10, 12–16]. The current participants of the Learning Collaborative include 21 programs and their health systems, often federally qualified health centers. These health systems are safety net providers who offer culturally competent primary care services for people on a sliding fee scale. [17] The CRCCP Learning Collaborative has explored many topics since its inception [18–26], and in the past few years, the theme of integrated delivery of interventions and supporting activities has been in the forefront of the discussions raised by health system participants. Health systems adopted approaches to integrate delivery of interventions and supporting activities to promote uptake of multiple cancer screenings. For example, we learned through conversations with CRCCP programs that health systems issued joint patient reminders for multiple cancer screenings or provider assessment and feedback reports for all cancer screenings, driven by an overall desire to identify synergies and efficiencies to enable them to sustain the interventions and supporting activities (Tangka FKL, Hoover S, Cariou C, Creighton B, Hobbs L, Marzano A, et al: Improving the efficiency of integrated cancer screening delivery across multiple cancers: case studies from Idaho, Rhode Island, and Nebraska, in preparation). This need for a more integrated approach was further amplified by the COVID-19 pandemic, as in-person patient contact was limited and offering one-stop-shop screening approach for preventive care could be a more efficient approach [27]. The effectiveness and cost-effectiveness of these approaches, though, have not been systematically assessed, and this lack of focused evaluation of integration highlights missed opportunities to optimize the promotion and delivery of cancer screening. Additionally, documenting these integrated approaches can offer useful lessons for other health systems and programs to identify opportunities to improve their processes.

Our goals are to describe the integrated approaches adopted by health systems to support joint implementation of evidence-based interventions and supporting activities to increase screenings for multiple cancers. First, we provide a conceptual model to formalize the levels and key areas of integration along the cancer continuum to offer a systematic framework to guide evaluation of integrated programs. Next, we present determinants of successful implementation of interventions, highlight process measurement challenges in evaluating integrated approaches, and suggest some implementation science research priorities to optimize joint delivery of interventions and supporting activities for increasing cancer screenings.

## Methods

We conducted an exploratory assessment using a mixed methods approach to examine integrated implementation of interventions and supporting activities to jointly increase screenings for colorectal, breast, and cervical cancer. In 2018, we began learning details about the integrated innovations that were occurring in the implementation settings through discussion and feedback during CRCCP Learning Collaborative webinars and conference calls with representatives from health systems and their partners, state health departments, and primary care associations.

First, we collected qualitative data from the programs’ implementation and evaluation plans. This information was used to develop key themes that were further explored during 10 site visits in 2018 and 2019 with health centers and partner organizations. Group discussions were held during these in-person visits using a site visit guide (see additional file 1) that contained key themes, questions, and probes related to cancer screening, integrated interventions, and other colorectal screening related topics. We allowed for structured and unstructured conversation to explore additional innovations. Two research team members were present for each site visit and captured detailed notes on the discussion. These notes were later reviewed to identify key themes for the conceptual framework, to identify process measurement challenges, and to generate suggested research areas. Furthermore, to develop the conceptual framework, we also reviewed previously developed cancer screening process models to identify whether an existing model could be updated to include integration [28–31]. None of the models fully captured the integration of interventions and supporting activities nor the integrated delivery of cancer screenings during a single visit and, therefore, we created a tailored model focused on integrated approaches.

Second, we then reviewed the most recent colorectal cancer, breast cancer, and cervical cancer screening recommendations of the United States Preventive Services Task Force (USPSTF), [1–3] which is provided in tabular format by cancer type, age, gender, frequency, and locations of screening services. This helped us to identify the extent to which eligible age groups and screening tests facilitated the implementation of joint cancer screenings.

Third, in the spring of 2019, RTI interviewed and polled six CRCCP programs about factors they believed to be most important in determining implementation success of interventions to increase cancer screening. We used all domains and constructs from the Consolidated Framework for Implementation Research (CFIR) [32] to query respondents. The CFIR constructs provide a tested approach to categorize potential determinants of implementing interventions and supporting activities. The domains included the following: intervention characteristics, outer setting, inner setting, individual characteristics, and process. Each domain contains several constructs; for example, inner setting constructs include structural characteristics and implementation climate. Respondents were provided the definition of each construct to ensure a common understanding of the constructs, and they were asked to rank each construct as either highly important, somewhat important, or of low importance. Each rank was given a numerical value, with 3 being most important and 1 being least important; we present the average of each construct across programs. In addition to these rankings, programs also provided feedback on other factors not fully captured in the CFIR constructs and we have summarized and categorized them into key thematic areas.

## Results

### Conceptual framework

**Fig. 1:**
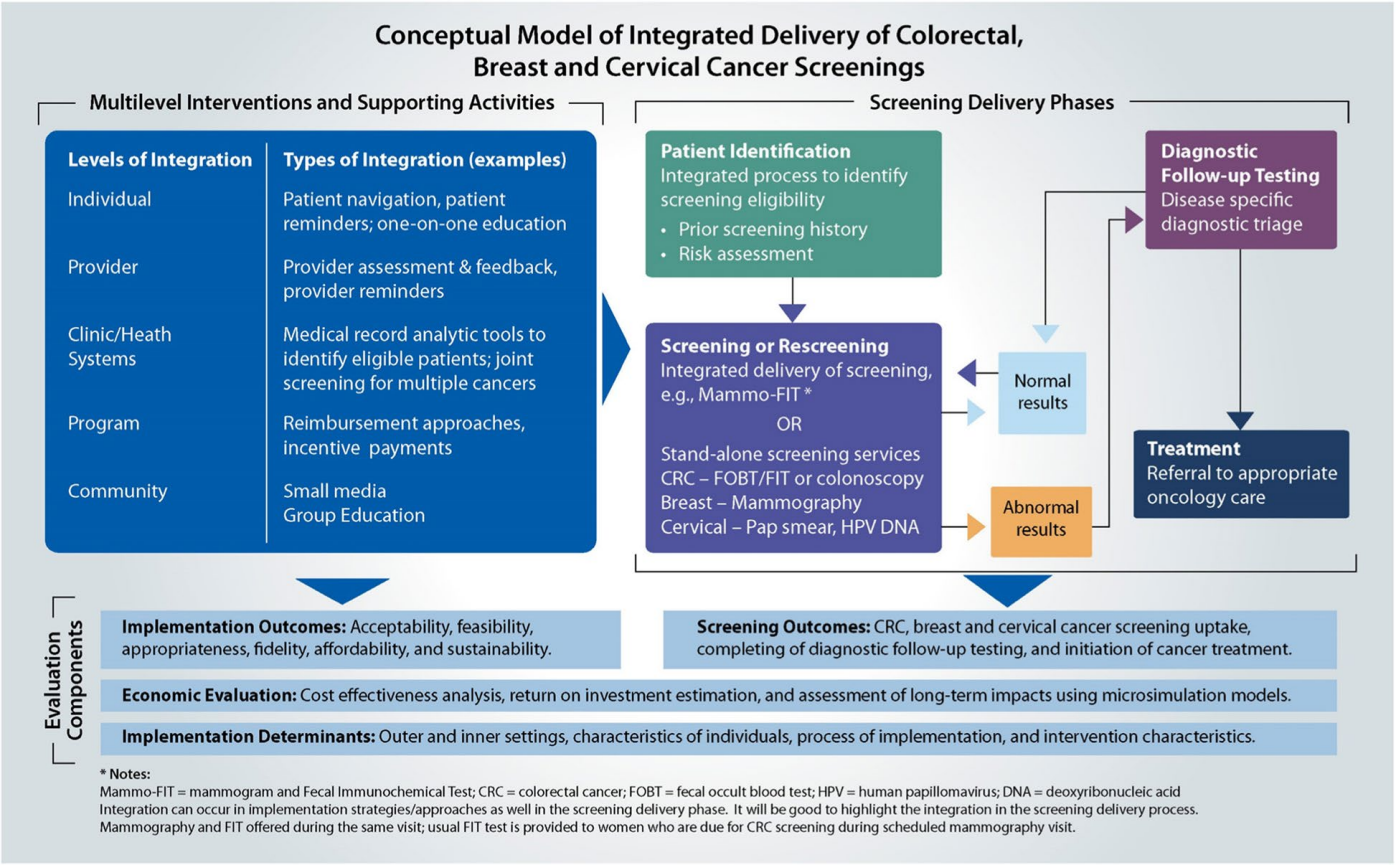
Conceptual framework

The conceptual model consists of three major categories (Fig. 1): (1) multilevel interventions and supporting activities; (2) screening delivery phases; and (3) evaluation components. Integration is possible to a different extent in each of these categories, as explored in detail below.

#### Multilevel integration of interventions and supporting activities

The integration of interventions and supporting activities can occur at all levels: individual, provider, health system, program, and community. Examples at the individual level include patient navigation, patient reminders, and one-on-one education; those at the provider level include provider assessment feedback and provider reminders. At the clinic or health system level, adaptations could be made to the electronic medical record systems to coordinate and track the screening process.

Integration often occurs across multiple levels. For example, at the program level, blended funding across colorectal, breast, and cervical cancers is provided to health systems. Integration in interventions occurs at the individual level for patient reminders and at the provider level with alerts through, for example, flags in the electronic medical record system. Further, programs can provide a single funding stream of support or reimbursement for support services delivered and can offer incentives that are aligned with screening goals across all the three cancers. In the community, small media and group education can be designed to address all three cancers instead of each individually. Health systems may also choose only to use integrated interventions and supporting activities for screening delivery, while others may implement some integrated processes (e.g., patient tracking systems and navigation support) for diagnostic follow-up testing and treatment initiation.

Table 1 provides examples of integrated evidence-based interventions and supporting activities implemented across selected CRCCP programs and health systems. Virginia integrated colorectal, breast, and cervical cancer screening interventions at the individual level by using patient navigation to promote all three screenings at once, when applicable. Similarly, Washington State implemented patient reminders across all three screenings. Nebraska and Rhode Island both made changes at the program level using contract vehicles to integrate colorectal, breast, and cervical cancer screening promotion activities. In Rhode Island, many of the health systems also integrated their patient navigation services to address all cancer screenings a person was eligible for during phone calls. Idaho chose to implement a variety of interventions at multiple levels. For example, partner health systems initially implemented evidence-based interventions and supporting activities for colorectal cancer screening and later added on breast and cervical cancer screenings as well.

**Table 1:** Integrated models among a select group of CRCCP programs and health systems

Level of integration	CRCCP program	Description of integration
Individual	Virginia Department of Health	Patient navigation was implemented across all three cancer screenings
Individual	Washington State Department of Health	Colorectal, breast and cervical cancer screening reminders were integrated into the patient reminder process
Individual and health system levels	West Virginia University	Patients received individualized reminders for one, two, or all three types of cancer screening, depending on which ones they were eligible to receive. Streamlined patient reminder process at the health system level for colorectal, breast, and cervical cancer screening were also developed.
Program	Nebraska Department of Health and Human Services	Joint subawards were used for all three cancer screenings. Eligible men and women in Nebraska were screened for colorectal cancer through the Nebraska Colon Cancer Program. Eligible women also received breast and cervical cancer screening through the NBCCEDP.
Program and individual level	Rhode Island Department of Health	All colorectal, breast, and cervical cancer screening funding to eight health systems was each under one contract, and patient navigation (in many instances for all cancers) was implemented across the health systems
Multiple levels	Idaho Department of Health & Welfare	Multilevel evidence-based interventions for colorectal cancer and often for breast and cervical cancer screenings were implemented in six health systems

#### Screening delivery phases

The first step in the screening process is to identify the person eligible for screening (e.g., gender and age), determine the patient’s prior screening history (e.g., has patient been screened before, date of prior screening), and assess the patient’s risk for the disease (e.g., family history of cancer). An essential step in this process is creating automated reports to quickly determine who is due for which screening tests. Some health systems are able to use their electronic medical records and overlay population health management modules to automate patient identification, whereas others still may require some manual review procedures. Regardless of the process selected, patient identification across all three cancer screenings can be generally integrated.

Patients are then informed by their health centers (through patient reminder phone calls or letters, for example) which cancer screenings they are eligible to receive. The screening delivery phases continue depending on the outcome of the screening test. If the patient’s test results are normal, the patient will be screened next based on USPSTF’s recommended timeframes. If the patient’s test results are abnormal, the patient will be referred for follow-up diagnostic testing and then cancer treatment when needed.

Integration can occur at various steps. Health systems and cancer screening programs may have stand-alone screening services for colorectal, breast, and cervical cancers for initial and repeat testing or, alternatively, some may combine screening tests. One example is Mammo-FIT (mammogram + fecal immunochemical test) where women who have an appointment for mammography for breast cancer screening receive a take-home FIT kit to screen for colorectal cancer, which can be returned via mail. A second example is programs that provide FIT kits during vaccination visits for COVID-19 and/or flu [33, 34].

Thus, we see that integration is possible to an extent and integration of interventions can support the delivery of joint screenings. But as shown in Table 2, the USPSTF [1–3] Grade A and B recommendations for routine colorectal, breast, and cervical cancer screenings are not uniform across factors, such as age, gender, and screening interval. The age ranges show an overlap of about 25 years for breast and colorectal screenings, but only for 15 years (ages 50–65) across all three cancer screenings. Although all three cancer screenings are applicable to women, only colorectal cancer screening is applicable to both men and women. And the frequency of screening test use and location of where testing takes place (e.g., at home, medical office, medical facility) varies, which can make it challenging to coordinate screening across all three cancers. Diagnostic testing and treatment referrals are usually individualized based on the patient needs, but the process of patient tracking, frequency of reminders, and navigation support can be standardized.

**Table 2:** USPSTF colorectal, breast, and cervical cancer screening recommendations

	**Colorectal (1)**	**Breast (2)**	**Cervical (2)**
Age range (1, 2)	45–75	50–74	21–65
Gender relevance (1, 2)	Male and female	Female	Female
Frequency of screening (1, 2)	Depends on test (annually to every 10 years)	Biennially	Depends on age and test (every 3–5 years)
Location of screening services	Stool tests—home tests distributed at office or mailed Colonoscopy—specialized service usually not available at health system	Mammography center not usually at health system	Pap/HPV DNA usually available at health system

#### Evaluation components

The implementation outcomes pertaining to integrated interventions and supporting activities will likely have to be jointly assessed, as it will be difficult to tease out issues specific to cancer screening. Qualitative assessment can be used to understand challenges or facilitators specific to a type of cancer screening. On the other hand, it will be possible to track screening outcomes separately for each cancer to assess potentially different impacts of the evidence-based interventions and supporting activities. Further evaluations to determine drivers of implementation success and economic evaluation could be conducted jointly on all cancers. The levels and type of integration selected may impact not only the cancer screening uptake, but also the cost and cost-effectiveness.

### Determinants of implementation success

**Fig. 2:**
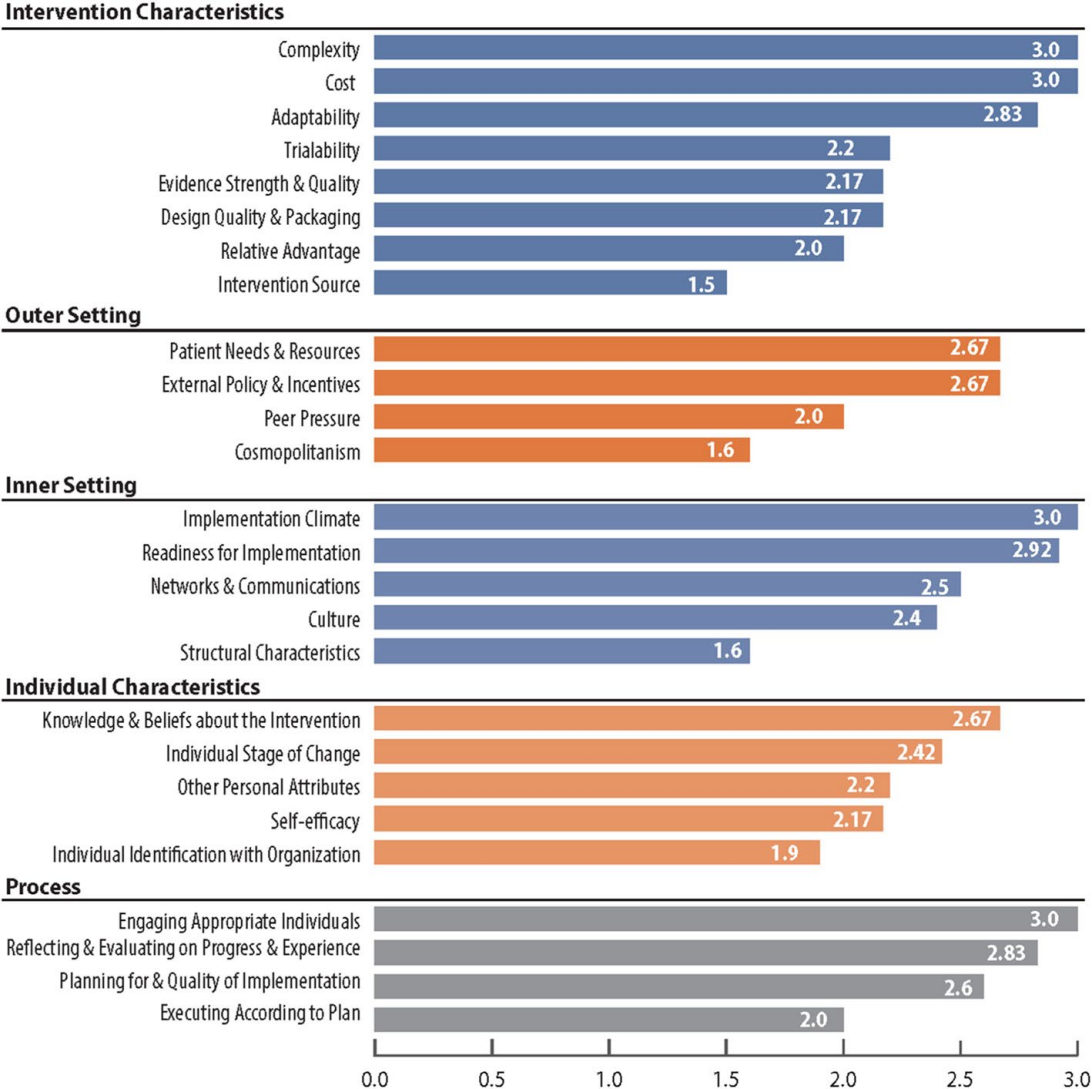
Programs’ ranking of CFIR constructs in implementing cancer screening interventions

In Fig. 2, we present the results from the programs’ rankings (3 indicating highest impact and 1 indicating lowest impact) of the constructs of the CFIR domains. The key determinants of successful implementation that were deemed to have the most impact (average score of 3.0) were complexity of the intervention, implementation cost, overall implementation climate, and ensuring appropriate individuals were involved in the implementation process. Higher levels of complexity and cost were negatively related to implementation success. Other factors that were also considered important (average score ranging from 2.6 to 2.9) were as follows: adaptability of the interventions and supporting activities; extent to which organization understood and prioritized patient needs; external incentives to support implementation efforts through payments and formal reporting requirements; readiness for implementation; clinic teams knowledge and beliefs about the intervention; ensuring appropriate procedures are in the place to review; and reflecting and evaluating implementation steps.

Overall, successful implementation reportedly involved many different factors, highlighting the complex nature of embedding interventions and supporting activities into existing workflow and competing clinic priorities. In addition to the specific CFIR constructs, respondents also indicated additional factors that were not fully captured in their rankings.

First, the size of clinic was an extremely important determinant of successful implementation, as larger clinics and health system have more staff members who can support each other in implementing the interventions.

Second, the overall structure of the health system—which may have multiple affiliated clinics—was critical; centralized or decentralized decision-making offered different levels of flexibility at the clinic level delivery of cancer screening. Centralized planning can be a facilitator as fewer resources are required at the clinic level for planning, but it also can be a barrier if there is no flexibility for adaptations.

Third, staff turnover was seen as a major barrier to both implementation success and sustainability. The burden consists of the need to train new staff, the loss of institutional knowledge, and the need to rebuild informal communication channels that are often required for coordination of multilevel interventions and supporting activities.

Fourth, the personal attributes of those implementing the interventions were important to consider and measure—motivation, competence, capacity, and resilience are key characteristics that drive success. These characteristics are included in a catch-all “other personnel attributes” category.

Fifth, the capacity and capabilities of the electronic medical records to support cancer screening by quickly identifying patients due for screening, tracking screening completion, and generating provider-level summary screening uptake were seen as critical for successful implementation and maintenance.

### Measurement challenges in evaluating effectiveness and cost

Evaluating multicomponent interventions is complex, as there can be differential impacts across the levels. There are also combined affects due to the implementation of multilevel interventions. Integration of these multilevel and multicomponent interventions and supporting activities can lead to further complexities in measurement and assessments, as there is integration across interventions with the shared goal of improving screening uptake for multiple cancers. Therefore, there are multiple intertwined interventions and supporting activities along with multiple screening outcomes.

Table 3 provides a listing of measurement challenges which are likely to impact process measures, clinical outcomes, and implementation outcomes. Additionally, there may be difficulties in estimating resource use and assessing economic impact, given challenges in assigning costs separately to each type of cancer screening, so only joint estimation of impacts may be feasible. Data capture approaches that can track activities and processes in detail may be valuable in conducting comprehensive evaluations. Conducting evaluations with a comparison group of clinics who only implement interventions and supporting activities for one cancer screening may provide important insights on the incremental effectiveness and cost.

**Table 3:** Measurement and evaluation challenges related to integrated implementation

	**Examples**	**Specific issues related to integration and measurement challenges**
Interventions and supporting activities	One-on-one education; patient reminders; provider reminders; electronic medical record system enhancements	Level of integration may vary across multicomponent interventions, with combined implementation at some levels and not others
Process measures	Proportion of patients receiving education; number of phone calls to remind patients or providers	Time spent during education sessions or phone calls on specific cancers may be difficult to accurately determine
Screening outcomes—short or medium term	Number of individuals screened; proportion completing recommended follow-up procedures	The denominator will differ for each type of screening based on individuals due for a screen during the time period of the intervention. Good tracking processes are required. Comparing outcomes across clinics may be challenging as individuals not up to date with screening may vary in the mix of screenings required during the intervention period. Diagnostic follow-up procedures are unique to each type of cancer screening and therefore the completion rates may also differ.
Screening outcomes—long term	Cancer mortality averted and life years saved due to screening	Long-term outcome estimates will require different microsimulation models for colorectal, breast, and cervical cancers. Joint impacts may be difficult to assign separately to each type of cancer screening
Implementation outcomes	Acceptability, appropriateness, feasibility, fidelity, sustainability	Joint implementation of interventions and supporting activities may mask implementation challenges related to each type of cancer screening
Cost measures and economic assessments	Activity-based cost of interventions; cost per successful screen	Stakeholders may not be able to separate out resource use and cost related to specific cancer screenings

However, there are challenges related to identifying appropriate comparator clinics, measuring the intensity of the interventions and supporting activities, and unpacking the differential impacts in screening uptake across the different cancers. Qualitative assessments using interviews and focus groups may provide invaluable information in identifying underlying differences in implementation processes across the three cancers, along with the facilitators and barriers that may differ.

## Discussion

We developed a conceptual framework to guide future implementation science-driven evaluation of integrated interventions and supporting activities for cancer screenings. We show that integration of interventions and supporting activities can occur at all levels: individual, provider, health system, program, and community. Furthermore, these integrated approaches can support joint delivery of cancer screenings such as Mammo-FIT programs where both breast and colorectal cancer screening are offered during a single visit. Integrated approaches, though, can pose measurement challenges, as it may be difficult to identify differential impacts of multilevel and multicomponent interventions on each type of cancer screening.

We found that the complexity of the intervention, cost of delivering the intervention and supporting activities, implementation climate at the clinic, and engaging appropriate team members in the implementation process were key determinants of implementation success. The implication of these findings for integration of interventions is not clearcut, as we anticipate that integration will likely increase complexity but can lead to cost efficiencies through synergies in the implementation process. Prior research studies have identified similar determinants with a recent study identifying screening processes embedded within clinic workflows and a culture of teamwork among the main facilitators for cancer screening [35]. Additional studies have also found that optimizing cancer screening processes by incorporating procedural steps within the electronic medical record system can support the cancer screening workflow [36, 37]. The interventions and screening pathways can be implemented in the electronic systems simultaneously for multiple cancer screenings, which can lead to savings in time and resources required to update the systems. Additionally, appropriate team members, such as champions and implementation leaders [38, 39], can be engaged in the screening process through joint trainings and quality improvement procedures for multiple cancer screenings.

To date, there is very limited research on integrated approaches; much of the focus has been on assessing single and multicomponent interventions and supporting activities for screening individual cancers [34, 40, 41]. A study on embedding stool testing along with flu shots concluded that the integrated approach was more effective than usual care in increasing colorectal cancer screening [34]. Another study showed that low-income women were more likely to undergo timely repeat mammograms when enrolled in both the cancer screening and chronic disease programs compared with the cancer screening program alone [41]. Historically, in the USA, most cancer screening initiatives have been implemented for single cancer screenings and this could be a reflection of the separate funding streams and incentives available. Health system and state-level payment incentives or quality metrics in the recent past have led to the prioritization of colorectal cancer screening uptake [23]. Health systems are attempting to leverage funding and process improvements related to colorectal cancer screening to also benefit breast and cervical cancer screenings through integration when feasible.

Conducting rigorous comparative assessments can yield important findings to ensure data-driven evidence is used to implement and optimize integrated screening for multiple cancers (and potentially other chronic conditions). Integrated approaches are being implemented in health systems under the hypothesis that integration will result in improved efficiencies. But we need a strong evidence-base on effectiveness and cost-effectiveness to ensure the limited resources that are available at health systems are appropriately focused on increasing cancer screenings among groups that have been economically and socially marginalized. We suggest the following research areas to address current gaps in our knowledge:

- (1) Understand patient preferences for integrated screenings to ensure that individuals eligible for screening are comfortable discussing or receiving information for multiple cancer screenings simultaneously; [42, 43]
- (2) Explore provider attitudes and capacity to deliver messaging related to multiple cancers and initiate referrals to complete cancer screenings individuals are eligible for; [44]
- (3) Assess health system or clinic capacity and readiness across a diverse group of varying sized facilities to implement interventions and supporting activities, offer high quality screenings, track multiple screenings, and provide support to individuals as needed; [45]
- (4) Study whether integrated implementation will lead to more widespread disruptions when changes in USPSTF recommendations require modification of process (e.g., change in frequency of cervical cancer screening or expanded age range for colorectal screening); [1, 2]
- (5) Quantify whether integrated approaches are likely to exacerbate overscreening for specific cancers (i.e., screening among persons for whom the risk outweighs potential benefits); [46, 47]
- (6) Evaluate the differential impacts on integrating screenings for multiple cancers when incentives or reporting metrics are focused on a single cancer (e.g., Oregon’s incentive metrics for coordinated care organizations to improve colorectal cancer screening from 2013 to 2019); [48]
- (7) Assess the efficiency gains of integration related to start-up (infrastructure investments and process modifications), ongoing implementation, and scale-up activities;
- (8) Conduct long-term studies to identify whether integrated interventions and supporting activities are more likely to be sustained compared to non-integrated cancer settings.

Importantly, health systems are currently using integrated approaches for implementing interventions and supporting activities for screening multiple chronic conditions in addition to cancer screenings. Therefore, the conceptualization and suggested future research areas presented in this manuscript have application beyond cancer screening. Evidence from comprehensive evaluations of integrated delivery of interventions and supporting activities will allow health systems serving population experiencing health disparities to make informed decisions to increase prevention and screening uptake for groups that have been marginalized.

## Conclusion

In this manuscript, we discuss the natural experiments that health systems are undertaking to implement integrated interventions and supporting activities for multiple cancer screenings, and, in some cases, for other chronic conditions as well. Integration to increase uptake of cancer screenings could be an effective and efficient approach, but we currently do not have the evidence to recommend widescale adoption. Systematic studies can help to ascertain the benefits of integration and best practices to optimize the delivery of integrated interventions and supporting activities to increase uptake of cancer screenings. We provide a conceptual model that highlights key determinants for consideration, review measurement challenges, and offer a list of priority research topics to galvanize the implementation science community to generate the required evidence.

## Supplementary Information

**Additional file 1.** CRCCP – Site Visit Guide

## Data Availability

Not applicable.

## Abbreviations

CDC: Centers for Disease Control and Prevention
CFIR: Consolidated Framework for Implementation Research
CRCCP: Colorectal Cancer Control Program
FIT: Fecal immunochemical test
NBCCEDP: National Breast and Cervical Cancer Early Detection Program
USPSTF: United States Preventive Services Task Force

## References

[1] Davidson KW, Barry MJ, Mangione CM, Cabana M, Caughey AB, U. S. Preventive Services Task Force (2021). Screening for colorectal cancer: us preventive services task force recommendation statement. JAMA.

[2] Curry SJ, Krist AH, Owens DK, Barry MJ, Caughey AB, U. S. Preventive Services Task Force (2018). Screening for cervical cancer: US preventive services task force recommendation statement. JAMA.

[3] Siu AL, Force USPST (2016). Screening for breast cancer: U.S. preventive services task force recommendation statement. Ann Intern Med..

[4] Sabatino SA, Thompson TD, White MC, Shapiro JA, de Moor J, Doria-Rose VP (2021). Cancer screening test receipt - United States, 2018. MMWR Morb Mortal Wkly Rep..

[5] Buskwofie A, David-West G, Clare CA (2020). A review of cervical cancer: incidence and disparities. J Natl Med Assoc.

[6] Demb J, Gupta S (2020). Racial and ethnic disparities in colorectal cancer screening pose persistent challenges to health equity. Clin Gastroenterol Hepatol.

[7] Hall IJ, Tangka FKL, Sabatino SA, Thompson TD, Graubard BI, Breen N (2018). Patterns and trends in cancer screening in the United States. Prev Chronic Dis.

[8] Seeff LC, DeGroff A, Tangka F, Wanliss E, Major A, Nadel M (2008). Development of a federally funded demonstration colorectal cancer screening program. Prev Chronic Dis.

[9] Joseph DA, DeGroff A (2019). The CDC Colorectal Cancer Control Program, 2009–2015. Prev Chronic Dis.

[10] Tangka FKL, Subramanian S, Hoover S, Lara C, Eastman C, Glaze B (2019). Identifying optimal approaches to scale up colorectal cancer screening: an overview of the centers for disease control and prevention (CDC)'s learning laboratory. Cancer Causes Control.

[11] Community Preventive Services Task Force (2016). The guide to community preventive services.

[12] Subramanian S, Tangka FK, Hoover S, Degroff A, Royalty J, Seeff LC (2011). Clinical and programmatic costs of implementing colorectal cancer screening: evaluation of five programs. Eval Program Plann.

[13] Subramanian S, Tangka FK, Hoover S, Beebe MC, DeGroff A, Royalty J (2013). Costs of planning and implementing the CDC's Colorectal Cancer Screening Demonstration Program. Cancer.

[14] Tangka FKL, Subramanian S, Beebe MC, Hoover S, Royalty J, Seeff LC (2013). Clinical costs of colorectal cancer screening in 5 federally funded demonstration programs. Cancer.

[15] Subramanian S, Tangka FKL, Hoover S, Royalty J, DeGroff A, Joseph D (2017). Costs of colorectal cancer screening provision in CDC's Colorectal Cancer Control Program: Comparisons of colonoscopy and FOBT/FIT based screening. Eval Program Plann.

[16] Tangka FKL, Subramanian S, Hoover S, Royalty J, Joseph K, DeGroff A (2017). Costs of promoting cancer screening: evidence from CDC's Colorectal Cancer Control Program (CRCCP). Eval Program Plann.

[17] Health Resources & Services Administration. Federally Qualified Health Centers [Internet]. What is a Health Center? Official web site of the U.S. Health Resources & Services Administration; 2021 [updated August [Available from: https://bphc.hrsa.gov/about/what-is-a-health-center/index.html.

[18] Lara CL, Means KL, Morwood KD, Lighthall WR, Hoover S, Tangka FKL (2018). Colorectal cancer screening interventions in 2 health care systems serving disadvantaged populations: Screening uptake and cost-effectiveness. Cancer.

[19] Tangka FKL, Subramanian S, Hoover S, DeGroff A, Joseph D, Wong FL (2020). Economic evaluation of interventions to increase colorectal cancer screening at federally qualified health centers. Health Promot Pract.

[20] Dacus HLM, Wagner VL, Collins EA, Matson JM, Gates M, Hoover S (2018). Evaluation of patient-focused interventions to promote colorectal cancer screening among New York state medicaid managed care patients. Cancer.

[21] Kemper KE, Glaze BL, Eastman CL, Waldron RC, Hoover S, Flagg T (2018). Effectiveness and cost of multilayered colorectal cancer screening promotion interventions at federally qualified health centers in Washington State. Cancer.

[22] Kim KE, Randal F, Johnson M, Quinn M, Maene C, Hoover S (2018). Economic assessment of patient navigation to colonoscopy-based colorectal cancer screening in the real-world setting at the University of Chicago Medical Center. Cancer.

[23] Barajas M, Tangka FKL, Schultz J, Tantod K, Kempster YM, Omelu N (2020). Examining the effectiveness of provider incentives to increase CRC screening uptake in neighborhood healthcare: a California Federally Qualified Health Center. Health Promot Pract.

[24] Conn ME, Kennedy-Rea S, Subramanian S, Baus A, Hoover S, Cunningham C (2020). Cost and effectiveness of reminders to promote colorectal cancer screening uptake in rural federally qualified health centers in West Virginia. Health Promot Pract.

[25] Hardin V, Tangka FKL, Wood T, Boisseau B, Hoover S, DeGroff A (2020). The effectiveness and cost to improve colorectal cancer screening in a federally qualified homeless clinic in Eastern Kentucky. Health Promot Pract.

[26] Kim KE, Tangka FKL, Jayaprakash M, Randal FT, Lam H, Freedman D (2020). Effectiveness and cost of implementing evidence-based interventions to increase colorectal cancer screening among an underserved population in Chicago. Health Promot Pract.

[27] Soloe C, Arena L, Schlueter D, Melillo S, DeGroff A, Tangka F, et al. Integrated implementation of evidence-based interventions to increase colorectal cancer screening through public health-primary care partnerships. Implement Sci Commun. Manuscript in review. 10.21203/rs.3.rs-127902/v1

[28] Taplin SH, Anhang Price R, Edwards HM, Foster MK, Breslau ES, Chollette V (2012). Introduction: understanding and influencing multilevel factors across the cancer care continuum. J Natl Cancer Inst Monogr.

[29] Zapka JG, Taplin SH, Solberg LI, Manos MM (2003). A framework for improving the quality of cancer care: the case of breast and cervical cancer screening. Cancer Epidemiol Biomarkers Prev.

[30] Beaber EF, Kim JJ, Schapira MM, Tosteson AN, Zauber AG, Geiger AM (2015). Unifying screening processes within the PROSPR consortium: a conceptual model for breast, cervical, and colorectal cancer screening. J Natl Cancer Inst.

[31] Subramanian S, Hoover S, Tangka FKL, DeGroff A, Soloe CS, Arena LC (2018). A conceptual framework and metrics for evaluating multicomponent interventions to increase colorectal cancer screening within an organized screening program. Cancer..

[32] Damschroder LJ, Aron DC, Keith RE, Kirsh SR, Alexander JA, Lowery JC (2009). Fostering implementation of health services research findings into practice: a consolidated framework for advancing implementation science. Implement Sci.

[33] Potter MB, Somkin CP, Ackerson LM, Gomez V, Dao T, Horberg MA (2011). The FLU-FIT program: an effective colorectal cancer screening program for high volume flu shot clinics. Am J Manag Care.

[34] Potter MB, Walsh JM, Yu TM, Gildengorin G, Green LW, McPhee SJ (2011). The effectiveness of the FLU-FOBT program in primary care a randomized trial. Am J Prev Med.

[35] Lam H, Quinn M, Cipriano-Steffens T, Jayaprakash M, Koebnick E, Randal F (2021). Identifying actionable strategies: using Consolidated Framework for Implementation Research (CFIR)-informed interviews to evaluate the implementation of a multilevel intervention to improve colorectal cancer screening. Implement Sci Commun.

[36] Nelson-Brantley H, Ellerbeck EF, McCrea-Robertson S, Brull J, Bacani McKenney J, Greiner KA, et al. Implementation of cancer screening in rural primary care practices after joining an accountable care organisation: a multiple case study. Fam Med Community Health. 2021;9(4). Available from: https://www.ncbi.nlm.nih.gov/pubmed/34937796. 10.1136/fmch-2021-001326 10.1136/fmch-2021-001326 PMC8710423 34937796

[37] Schlueter D, DeGroff A, Soloe C, Arena L, Melillo S, Tangka F, et al. Factors that support sustainability of health systems change to increase colorectal cancer screening in primary care clinics: a longitudinal qualitative study. Health Promot Pract. 2022:15248399221091999. Available from: https://www.ncbi.nlm.nih.gov/pubmed/35582930. 10.1177/15248399221091999 10.1177/15248399221091999 PMC9672135 35582930

[38] Leeman J, Askelson N, Ko LK, Rohweder CL, Avelis J, Best A (2020). Understanding the processes that Federally Qualified Health Centers use to select and implement colorectal cancer screening interventions: a qualitative study. Transl Behav Med.

[39] Liang S, Kegler MC, Cotter M, Emily P, Beasley D, Hermstad A (2016). Integrating evidence-based practices for increasing cancer screenings in safety net health systems: a multiple case study using the Consolidated Framework for Implementation Research. Implement Sci.

[40] Tabung FK, Daguise VG, Lydiard D, Steck SE (2017). An integrated approach to addressing chronic disease risk factors in financially disadvantaged women in South Carolina. Am J Health Promot.

[41] Gregory-Mercado KY, Will J, True S, Royalty J, Starcher ET, Khavjou O (2007). A combined approach to women's health is associated with a greater likelihood of repeat mammography in a population of financially disadvantaged women. Prev Chronic Dis.

[42] Brandzel SD, Bowles EJA, Wieneke A, Bradford SC, Kimbel K, Gao H (2017). Cancer screening reminders: addressing the spectrum of patient preferences. Perm J.

[43] Buist DSM, Gao H, Anderson ML, Onega T, Brandzel S, Rabelhofer MA (2017). Breast cancer screening outreach effectiveness: mammogram-specific reminders vs. comprehensive preventive services birthday letters. Prev Med.

[44] Neugut AI, MacLean SA, Dai WF, Jacobson JS (2019). Physician characteristics and decisions regarding cancer screening: a systematic review. Popul Health Manag.

[45] Miake-Lye IM, Delevan DM, Ganz DA, Mittman BS, Finley EP (2020). Unpacking organizational readiness for change: an updated systematic review and content analysis of assessments. BMC Health Serv Res.

[46] Moss JL, Roy S, Shen C, Cooper JD, Lennon RP, Lengerich EJ (2020). Geographic variation in overscreening for colorectal, cervical, and breast cancer among older adults. JAMA Netw Open.

[47] Kepka D, Breen N, King JB, Benard VB, Saraiya M (2014). Overuse of papanicolaou testing among older women and among women without a cervix. JAMA Intern Med.

[48] Oregon Health Authority. CCO incentive measure specification sheet for 2019 measurement year: colorectal cancer screening, basic health measurement. Oregon Health Authority; 2018 [Available from: https://www.oregon.gov/oha/HPA/ANALYTICS/CCOMetrics/2019-Colorectal-Cancer-Screening.pdf.

