# Supplementary material for: Integrated interventions and supporting activities to increase uptake of multiple cancer screenings: conceptual framework, determinants of implementation success, measurement challenges, and research priorities
Source: Implement Sci Commun. 2022 Oct 5;3:105. doi: 10.1186/s43058-022-00353-8 (PMC9532830; doi:10.1186/s43058-022-00353-8)
Supplement: Supplementary file 1 — Additional file 1. CRCCP – Site Visit Guide [file 43058_2022_353_MOESM1_ESM.docx]

Additional file 1. CRCCP – Site Visit Guide

**Part 1: Partners, health system and clinics**

1. Who are the main partners?
2. Which health system is involved? More than one?
3. What types of clinics and geographic areas are included?
4. How many clinics overall? How many targeted now and in the future?
5. Are all clinics in the health system participating in the planned interventions?

**Part 2: Target population**

1. Which group of individuals was selected? Why was this group selected?
2. What is the baseline colorectal cancer screening compliance rate for this group? How was it measured (chart review, EHR)? What is your level of confidence on the accuracy of the rate?
3. Does the baseline colorectal cancer screening compliance rate vary by clinic?

**Part 3: Planned intervention**

1. What are the planned interventions? What already exists in the health system? Can we get details on the features of the provider reminders, client reminders and other interventions?
2. Which outcomes are targeted (screening compliance, diagnostic compliance, timeliness of treatment)?
3. What colorectal cancer screening test will be used? Only including average risk? What about those who are symptomatic?
4. Who will be paying for the screening, diagnostic and required procedures?
5. What were the lessons learned through the needs assessment? What are the facilitators and barriers for implementing the interventions?
6. What about capacity to perform colonoscopies?
7. What level of improvement (increase in colorectal compliance rate) do you expect?
8. What are the roles of the different partners? Who is designing intervention?
9. Who is implementing the intervention? What procedures will be followed to ensure Evidence-based interventions are implemented as planned? Who will be monitoring the sites?

**Part 4: Integration with other screenings or programs**

1. Which cancer screenings interventions are delivered jointly (e.g., colorectal cancer screening along with breast cancer screening patient reminders)?
2. Are interventions jointly delivered for other chronic disease programs in addition to cancer screening (e.g., hypertension screening).
3. What types of interventions and supporting activities are integrated?
4. When did you initiate the integration of interventions and supporting activities?

**Part 5: Planned evaluation**

1. What types of evaluation are you planning to do? In addition to CDC mandates?
2. What types of data collection is planned? Qualitative? Quantitative?
3. Will you be collecting additional patient level data? On barriers?
4. Are there any barriers that you foresee in collecting information on the cost of the interventions?

**Part 6: Selection of clinics**

1. Are there clinics that are similar to the ones in which the interventions will be implemented?
2. Are these within the same health system? Geographic area?
3. Do they target the same population? Age group? Racial mix? Education level? Insurance status?
4. What types of interventions are currently implemented at these potential comparison clinics?
5. Can we get the baseline colorectal cancer screening compliance rate for the proposed comparison clinic?

**Part 7: Timeline and procedures**

1. When will the intervention be implemented? How many sites are targeted initially?
2. What is the timeline for assessing the outcome of the intervention?

**Part 8: Other topics**
